# Exosomal Mir-3613-3p derived from oxygen–glucose deprivation-treated brain microvascular endothelial cell promotes microglial M1 polarization

**DOI:** 10.1186/s11658-023-00432-1

**Published:** 2023-03-05

**Authors:** Mengqi Zhang, Qian Wu, Mimi Tang, Zhuohui Chen, Haiyue Wu

**Affiliations:** 1 Department of Neurology, Xiangya Hospital, Central South University, 87 Xiangya Road, Changsha, 410008 Hunan China; 2 National Clinical Research Center for Geriatric Disorders, Xiangya Hospital, Central South University, Changsha, 410008 China; 3 Department of Neurology, First Affiliated Hospital, Kunming Medical University, Kunming, 650032 China; 4 Department of Pharmacy, Xiangya Hospital, Central South University, Changsha, 410008 China; 5 Institute of Hospital Pharmacy, Xiangya Hospital, Central South University, Changsha, 410008 China

**Keywords:** Exosome, Ischemic stroke, Brain microvascular endothelial cell, Macrophage, Neuron

## Abstract

**Background:**

Brain microvascular endothelial cell (BMEC) injury can affect neuronal survival by modulating immune responses through the microenvironment. Exosomes are important vehicles of transport between cells. However, the regulation of the subtypes of microglia by BMECs through the exosome transport of microRNAs (miRNAs) has not been established.

**Methods:**

In this study, exosomes from normal and oxygen–glucose deprivation (OGD)-cultured BMECs were collected, and differentially expressed miRNAs were analyzed. BMEC proliferation, migration, and tube formation were analyzed using MTS, transwell, and tube formation assays. M1 and M2 microglia and apoptosis were analyzed using flow cytometry. miRNA expression was analyzed using real-time polymerase chain reaction (RT-qPCR), and IL-1β, iNOS, IL-6, IL-10, and RC3H1 protein concentrations were analyzed using western blotting.

**Results:**

We found that miR-3613-3p was enriched in BMEC exosome by miRNA GeneChip assay and RT-qPCR analysis. miR-3613-3p knockdown enhanced cell survival, migration, and angiogenesis in the OGD-treated BMECs. In addition, BMECs secrete miR-3613-3p to transfer into microglia via exosomes, and miR-3613-3p binds to the *RC3H1* 3′ untranslated region (UTR) to reduce RC3H1 protein levels in microglia. Exosomal miR-3613-3p promotes microglial M1 polarization by inhibiting RC3H1 protein levels. BMEC exosomal miR-3613-3p reduces neuronal survival by regulating microglial M1 polarization.

**Conclusions:**

miR-3613-3p knockdown enhances BMEC functions under OGD conditions. Interfering with miR-3613-3p expression in BMSCs reduced the enrichment of miR-3613-3p in exosomes and enhanced M2 polarization of microglia, which contributed to reduced neuronal apoptosis.

**Supplementary Information:**

The online version contains supplementary material available at 10.1186/s11658-023-00432-1.

## Background

Stroke, with ischemic stroke as the primary subtype. It is characterized by high morbidity, mortality, disability, and recurrence rates. It is one of the three major diseases that cause human death [1]. From 1990 to 2019, the incidence of ischemic stroke in China increased significantly, which brought a heavy burden to society and patients [2, 3]. Neuron loss occurs after ischemic stroke, which affects the stability and integrity of brain function and directly leads to functional defects. Therefore, the protection and regeneration of neurons has been the main focus for the effective rescue of brain functional deficits. Tissue-type plasminogen activator is currently a widely accepted treatment and most effective when administered within 4–5 h of acute ischemic stroke; however, it is suitable for only a limited number of patients due to its strict time window [4]. Therefore, it is important to seek early and effective therapeutic strategies for neuronal protection and regeneration to improve the prognosis of ischemic stroke.

With the advancement of neuroscientific research, the neurovascular unit (NVU) has received increasing attention in recent years. The neurovascular unit is composed of neuron-gliocyte vessels, and all components are closely related in structure to maintain brain homeostasis [5]. Immediately after ischemic stroke, brain microvascular endothelial cells (BMECs) are damaged, resulting in severe damage to the blood–brain barrier [6–13]. Disruption of the vascular network leads to the interruption of local nerve tissue nutritional support, which is considered the key initiator of cerebral ischemic injury [14]. Studies have shown that the destruction of the vascular network structure and blood supply disorders caused by vascular dysfunction are important factors that trigger a neuroinflammation cascade, lead to ischemic necrosis of neurons, and aggravate neurological deficits. Promoting angiogenesis can effectively restore nerve tissue perfusion, improve cerebral ischemia, and promote the remodeling of neurovascular units [15]. Early rescue of the damaged microvascular system after stroke injury can effectively reduce the post-ischemic inflammatory response, thereby reducing brain injury [15]. The existence of microvessels can also promote the growth of axons into the injury site and, at the same time, inhibit the activation of astrocytes, promote axonal connections, and improve nerve function [16]. Therefore, it is of great significance to scientifically analyze the initiating factors of vascular network structure and function remodeling after ischemic stroke and actively seek early and effective neurovascular protection strategies to improve the prognosis of stroke.

Microglia are important immune cells in the central nervous system; they include M0, M1, and M2 subtypes. Studies have shown that, during the early stage of ischemic stroke, microglia are M2-polarized to promote neural repair and play a protective role. Under the influence of some external factors such as traumatic brain injury and focal transient cerebral ischemia, local microglia showed M2 phenotype at the beginning of cerebral ischemia which changed into the M1 phenotype over time., resulting in neuronal death [17, 18]. Inhibiting microglial M1 polarization and promoting and maintaining microglial M2 polarization can restore neuronal plasticity and improve neurological function in patients with ischemic stroke [19, 20]. BMECs are dynamic cells that respond to the extracellular microenvironment. Following changes in the external microenvironment, they communicate with microglia by producing various signal molecules, regulating the subtypes of microglia, and affecting tissue and organ damage repair [21, 22]. Therefore, it is important to further understand the regulation of microglial subtypes by BMECs after stroke.

Exosomes are an important communication medium between cells that can transport the loaded substances to the target cells and affect the biological functions of the target cells [23, 24]. Recent studies have shown that exosomal microRNAs (miRNAs) can be taken up by target cells and subsequently regulate their function; they play an important role in vascular and neural regeneration after stroke [25]. However, the regulation of subtypes of microglia by BMECs through the exosome transport of miRNAs has not been established.

In this study, we analyzed the expression profiles of miRNAs in BMEC exosomes using miRNA GeneChip assay and found that miR-3613-3p was enriched in BMEC exosomes. Next, we investigated the effect of miR-3613-3p on high-glucose-induced BMEC proliferation, migration, and angiogenesis and analyzed the regulatory effect, and its underlying mechanism, of BMEC exosomal miR-3613-3p on macrophage subtypes. Through the above studies, the role of miR-3613-3p in BMEC injury and microglial polarization was elucidated, providing a potential target for stroke treatment.

## Methods

### Cell culture, oxygen–glucose deprivation (OGD) treatment, and exosome extraction and treatment

Human brain microvascular endothelial cells (BMECs, Procell, Wuhan, China) were cultured and treated with OGD, as previously described [26]. OGD induction: BMECs were cultured in D-Hank’s medium to instead of normal culture medium and incubated under hypoxic conditions (temperature 37 °C, and atmosphere 95% N_2_ and 5% CO_2_), then exposed to OGD for 4 h. Exosomes from culture supernatants were collected from normally cultured and OGD-cultured BMECs using the Cell Supernatant Exosome Extraction Kit (Solarbio, Beijing, China). Overspeed centrifugal (Sorvall, ThermoScientific, Waltham, MA, USA) condition for exosome collection: 12,000*g*, 4 °C, 2 h. Next, 200 μg of exosomes were added to 50 μl of the PKH67 working solution (Umibio, Shanghai, China) and incubated for 10 min. HMC3 cell line (zqxzbio, Shanghai, China), a human microglial line, was cultured in MEM medium with 10% PBS and 1%P/S (zqxzbio) and incubated in humidified 5% CO_2_ and 95% air at 37 °C. After culturing for 24 h, 6.3 × 10^9^ particles per well of exosomes were added to MEM medium and co-cultured for 24 h. HMC3 cells were collected for M1 and M2 polarization and western blot analysis. Finally, human cortical neurons (Procell, Wuhan, China) were cultured in a complete culture medium of human cortical neurons (Procell). After a 24-h culture, the exosome-treated HMC3 cells (0.5 × 10^5^ cells per well) were cultured in the upper inserts of the Transwell chamber (0.4 μm pore size, Corning Life Sciences, Corning, NY, USA), and the cortical neurons (0.5 × 10^5^ cells per well) were cultured in the lower inserts. After co-culture for 48 h, the neurons were collected for proliferation and apoptosis assays.

### Exosome extraction and miRNA GeneChip assay

The BMECs were exposed to OGD and a normal culture medium for 4 h. Exosomes from the culture supernatants were collected using a Cell Supernatant Exosome Extraction Kit (Solarbio, Beijing, China), and RNA was extracted using TRIzol (Life Technologies, Carlsbad, CA, USA). The RNA concentrations were measured using a NanoDrop 2000 spectrophotometer (Thermo Electron Corporation, USA) at 260/280 nm and by subjecting RNA to a Bioanalyzer 4200 (Agilent, Santa Clara, CA, USA). GeneChip assay was performed by Affymetrix miRNA 4.0 GeneChip as previously described [27]. For microarray analysis, we used the limma package for screening differentially expressed miRNAs. The screening conditions were as follows: *p*-value of < 0.05, fold change of > 1.2 (significantly upregulated miRNA), or fold change of < 0.8333 (significantly downregulated miRNA). Differentially expressed miRNA-target interactions were analyzed using miRTarBase. The related functions, such as biological processes, cellular components, and molecular functions, and the pathways of target genes were analyzed using the Gene Ontology and Kyoto Encyclopedia of Genes and Genomes analysis [28–33].

### Quantitative real-time polymerase chain reaction assay

Quantitative real-time polymerase chain reaction (qRT-PCR) was used to evaluate the levels of miRNA expression. qRT-PCR was performed as previously described [26]. Briefly, total RNA was extracted using TRIzol reagent (Invitrogen, Carlsbad, CA, USA). First-strand cDNA was synthesized using the ImProm-II reverse transcription system (Promega, Madison, WI, USA). qRT-PCR was performed using the SYBR Premix ExTaq II kit (Takara, Dalian, China) on a 7500 Real-Time PCR System (Applied Biosystems; Thermo Fisher Scientific, Inc.). U6 was used as an endogenous control to normalize the miRNA expression. The relative expression levels of the miRNAs were calculated using the 2^−ΔΔCt^ method. All reactions were performed in triplicates. The primer sequences used are presented in Table 1.

**Table 1:** Primer sequences

Target	Primer sequences (5′–3′)
hsa-miR-1290-F	ACACTCCAGCTGGGTGGATTTTTGGATCAGGG
hsa-miR-8084-F	ACACTCCAGCTGGGGAATACTAAGTAAAA
hsa-miR-3201-F	ACACTCCAGCTGGGGGGATATGAAGAAAAA
hsa-miR-4668-5p-F	ACACTCCAGCTGGGAGGGAAAAAAAAAAGGA
hsa-miR-3613-3p-F	ACACTCCAGCTGGGACAAAAAAAAAAGCCCA
miRNA universal primer-R	CTCAACTGGTGTCGTGGA
U6-F	CTCGCTTCGGCAGCACA
U6-R	AACGCTTCACGAATTTGCGT

### Western blot assay

The microglia were collected, and protein was extracted for western blotting to detect the markers of M1 microglia, as well as the markers of M2 microglia, such as IL-10 and ring finger and CCCH-type domains 1 (RC3H1). Western blotting was performed as previously described [26]. The primary antibodies of CD86 (1/1000 dilution, ab239075), tubulin (1/5000 dilution, ab7291), IL-1β (1/1000 dilution, ab254360), iNOS (1/1000 dilution, ab178945), IL-6 (1/1000 dilution, ab233706), IL-10 (1/5000 dilution, ab133575), and RC3H1 (1/5000 dilution, ab70196) were purchased from Abcam (Cambridge, UK). The secondary antibodies were anti-rabbit antibodies conjugated with horseradish peroxidase (Southern Biotech, Birmingham, AL, USA). GAPDH was used as the internal reference.

### Cell transfection

miR-3613-3p inhibitor (20 μM) and miRNA negative control inhibitor (20 μM, NC inhibitor) (GenePharma, Suzhou, China) were transfected into BMECs (Invitrogen; Thermo Fisher Scientific, Inc.) using Lipofectamine RNAiMAX (Invitrogen, Carlsbad, CA, USA). siRNAs for *RC3H1* (si-*RC3H1*, 20 μM) and the negative control (si-NC, 20 μM) (GenePharma) were transfected into microglia using Lipofectamine 2000 (Invitrogen). Twenty-four hours after transfection, BMECs and microglia were cultured for further study.

### Proliferation, apoptosis, migration, tube formation assays, and ELISA

Cell proliferation was analyzed using the CellTiter 96 AQueous One Solution Cell Proliferation Assay kit (MTS, Promega, Madison, WI, USA). The Annexin V-FITC/PI apoptosis detection kit (Keygen, Nanjing, China) was used to determine the apoptosis rate of nerve cells, which analyzed by flow cytometer (BD Biosciences, San Jose, CA, USA). Experimental procedures of cell proliferation and apoptosis were made according to the manufacturer’s protocol. Migration and tube formation assays were performed as previously described [26]. Total number of meshes/nodes and branching/segments length in tube formation assay were analyzed by the Angiogenesis analyzer of imageJ 6.0 (National Institutes of Health, Bethesda, Maryland,USA). IL-1β (KA0356, Abnova, Taipei, Taiwan) and IL-10 (bsk11010, Bioss, Beijing, China) levels were measured by ELISA according to operating manual.

### Statistical analysis

All experiments were performed in triplicate. Data statistics and analyses were performed using SPSS software (version 19.0). Data conforming to a normal distribution are shown as mean ± standard deviation. Differences between the two groups were analyzed using Student’s *t*-test. Analysis of variance (ANOVA) was used for the overall comparison of the measurement indices between more than two groups, followed by post-hoc Dunnett’s multiple comparisons test. Differences were considered statistically significant (*).

## Results

### miR-3613-3p knockdown enhanced cell survival, migration, and angiogenesis in the OGD-treated BMECs

**Fig. 1:**
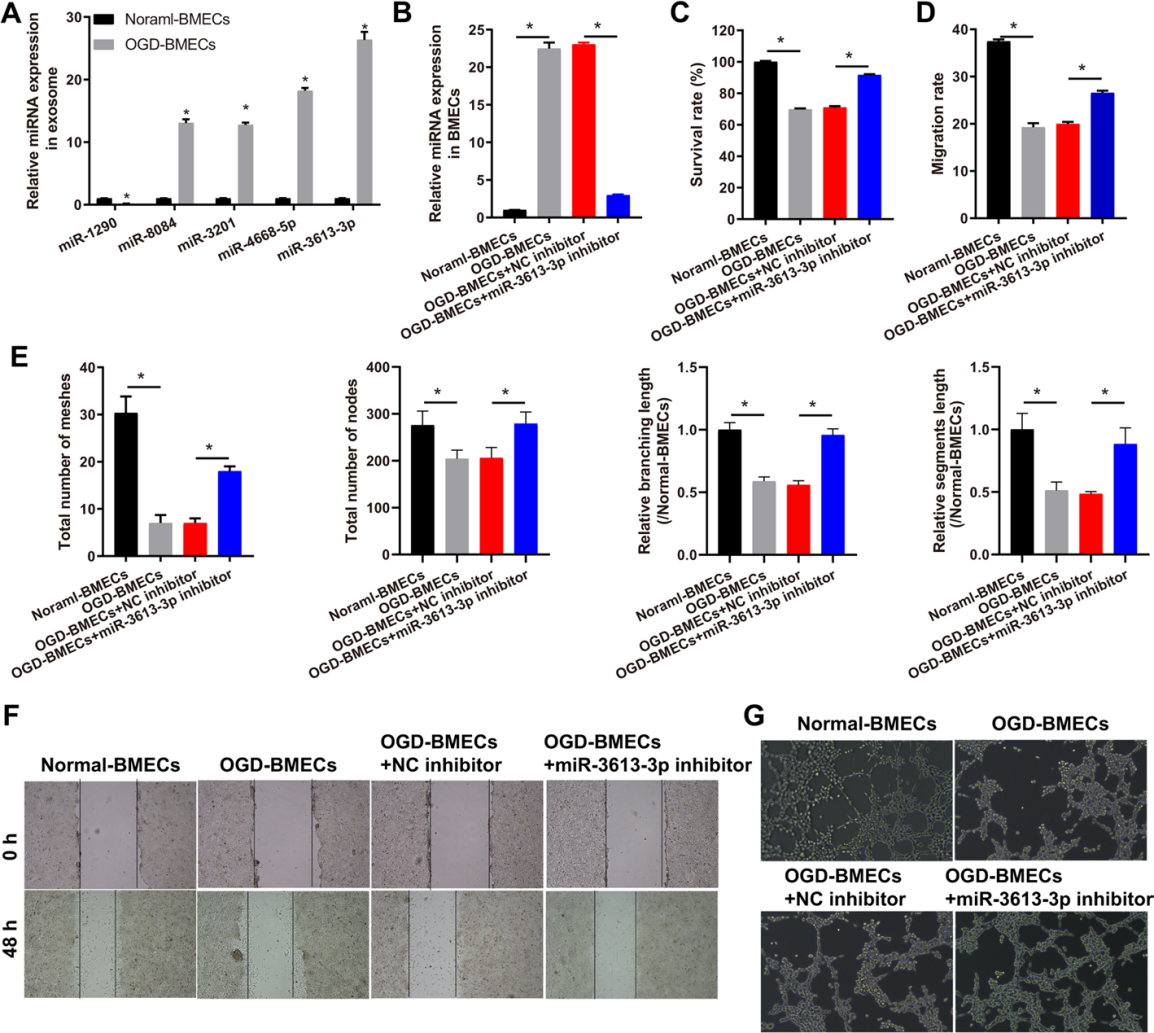
miR-3613-3p knockdown recovered BMEC proliferation, migration, and angiogenesis in OGD-treated BMECs. **A** Five miRNAs that prominently changed in the OGD-treated BMEC exosomes based on miRNA GeneChip assay were also confirmed to have prominently changed in the OGD-treated BMEC exosomes on the basis of RT-qPCR. Differences were analyzed using *t*-test. *P < 0.05. **B** miR-3613-3p expression in BMECs were measured by qRT-PCR after transfection at 24 h and treated for 4 h. **C** BMEC proliferation was measured by MTS, and the survival was calculated according to the percentage of the test group/control group (normal-BMECs group). **D**, **E** The bar represents the migration rate and angiogenesis (include total number of meshes/nodes and branching/segments length), respectively. **F**, **G** Representative image of the migration rate and angiogenesis after transfection at 48 h, followed by OGD treatment for 4 h. Differences were analyzed using ANOVA, followed by post-hoc Dunnett’s multiple comparisons test. *p < 0.05.

First, we collected exosomes from the culture medium of OGD-treated and normally treated BMECs and extracted miRNA for miRNA GeneChip assay. The exosome identification results showed that the isolated exosomes were membrane vesicles with a diameter of 30–100 nm, with high expression of the exosome marker CD81 and low expression of tubulin (Additional file 1: Fig. S1A–C). The results showed that 205 miRNAs were prominently reduced, while 49 miRNAs were prominently enriched (Additional file 1: Fig. S1D and E). The results of the Gene Ontology and Kyoto Encyclopedia of Genes and Genomes analyses are shown in Additional file 1: Fig. S2. Abnormally downregulated miRNAs were most associated with focal adhesion, transcriptional activator activity, endomembrane system organization, and pathways in cancer (Additional file 1: Fig. S2A and B), while abnormally upregulated miRNAs were most correlated with the transcription factor complex, ubiquitin-like protein transferase activity, positive regulation of cell cycle, and pathways in cancer (Additional file 1: Fig. S2C and D). To further verify the expression of exosome-derived miRNAs, we selected the five most aberrantly expressed miRNAs. The results showed that compared with exosomes from normal-treated BMECs, miR-1290 expression were prominently reduced (1 ± 0.01 versus 0.15 ± 0.01), whereas miR-8084 (1 ± 0.02 versus 13.07 ± 0.6), miR-3201 (1 ± 0.04 versus 12.76 ± 0.36), miR-4668-5p (1 ± 0.03 versus 18.21 ± 0.45), and miR-3613-3p (1 ± 0.04 versus 26.37 ± 1.23) expressions were prominently enriched in exosomes from OGD-treated BMECs (Fig. 1A). Since the effects of the above five miRNAs on stroke and macrophage polarization are not clear, the miRNAs whose expression had the greatest difference (miR-3613-3p) were selected to further verify the effect of exosomal miRNAs on NVU. To further verify the effect of miR-3613-3p on OGD-treated BMECs, miR-3613-3p was knocked down in BMECs and treated with OGD for 4 h (Fig. 1B). miR-3613-3p expression was prominently higher in OGD-treated than in normal BMECs (1.00 ± 0.020 versus 22.48 ± 0.800); OGD-treated BMECs showed lower expression after transfection with the miR-3613-3p inhibitor than the NC inhibitor group (23.05 ± 0.240 versus 2.97 ± 0.080). In addition, BMEC survival (100.00 ± 0.504 versus 69.82 ± 0.530), migration (37.49 ± 0.404 versus 19.28 ± 0.847), and angiogenesis [include total number of meshes (30.33 ± 3.512 versus 7.00 ± 1.732), total number of nodes (275.7 ± 30.010 versus 204.30 ± 18.610), branching length (1.00 ± 0.057 versus 0.59 ± 0.035), and segment length (1.00 ± 0.128 versus 0.51 ± 0.068)] were prominently lower in OGD-treated BMECs than in normal-treated BMECs, and the survival (71.03 ± 0.779 versus 91.65 ± 0.504), migration (19.97 ± 0.433 versus 26.56 ± 0.471), and angiogenesis [include total number of meshes (7.00 ± 1.000 versus 18.00 ± 1.000), total number of nodes (206.00 ± 22.00 versus 279.00 ± 25.24), branching length (0.56 ± 0.034 versus 0.96 ± 0.048), and segment length (0.49 ± 0.018 versus 0.88 ± 0.129)] of OGD-treated BMECs was prominently recovered after transfection with the miR-3613-3p inhibitor than the NC inhibitor group (Fig. 1C–G).

### BMEC exosomal miR-3613-3p is transferred into microglia

**Fig. 2:**
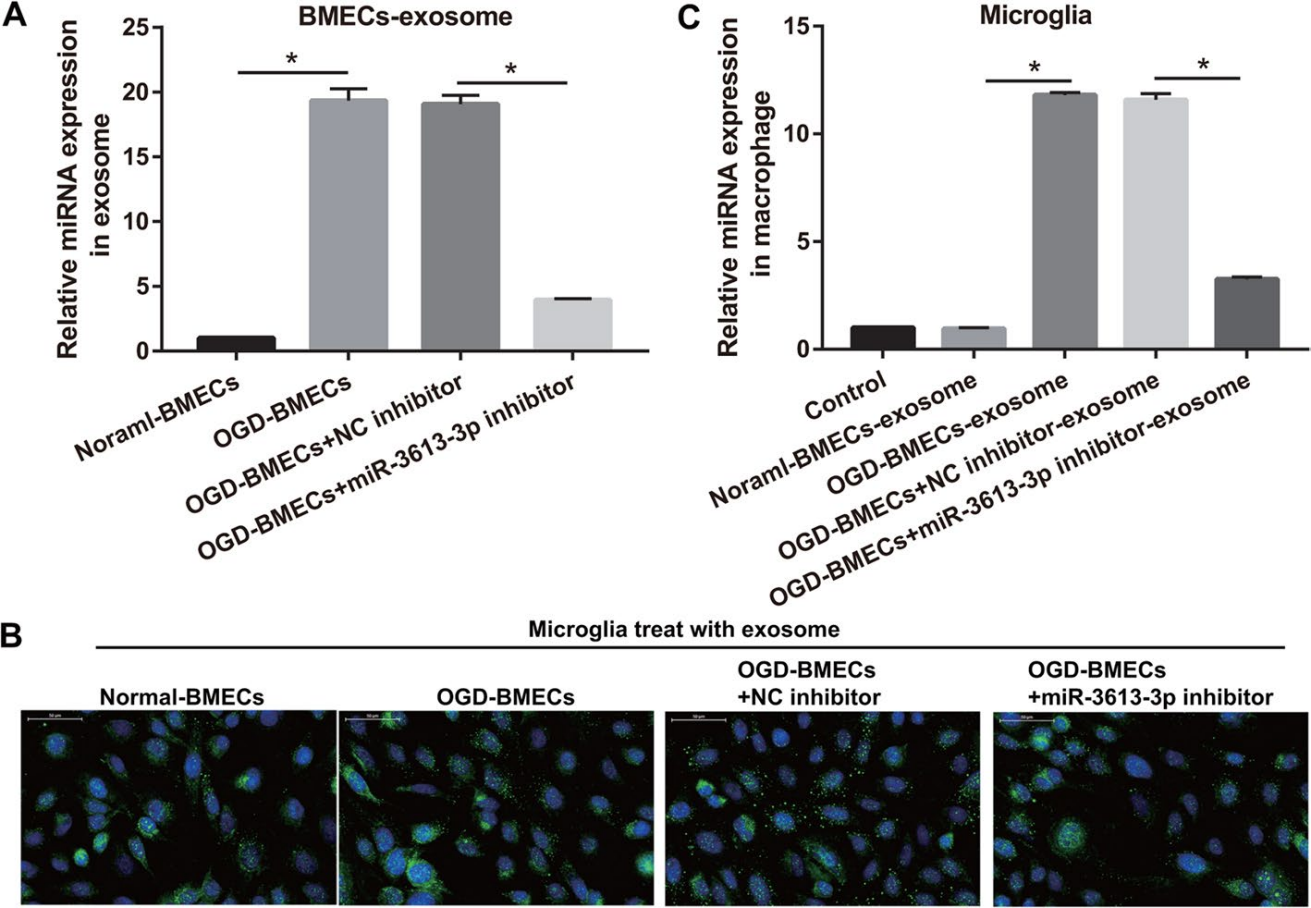
BMECs secrete miR-3613-3p to transfer into microglia via exosomes. **A** miR-3613-3p expression in BMEC exosome after transfection was detected by RT-qPCR. **B** HMC3s were treated with PKH 67 fluorescently labeled BMEC exosome, and the fluorescence was observed under a fluorescence microscope. Green, PKH 67 fluorescence. **C** miR-3613-3p expression in HMC3 after co-culturing with BMEC exosomes was detected by RT-qPCR. Differences were analyzed using ANOVA, followed by post-hoc Dunnett’s multiple comparisons test. *p < 0.05

To further understand the effect of BMEC exosomal miR-3613-3p on HMC3 polarization, we co-cultured exosomes with HMC3s. First, miR-3613-3p expression was significantly higher in the exosomes of OGD-treated BMECs (19.35 ± 0.92) than in the exosomes of normal-treated BMECs (1.00 ± 0.03); it was prominently reduced in the exosomes of OGD-treated BMECs after miR-3613-3p inhibitor transfection compared with NC inhibitor transfection (3.95 ± 0.10 versus 19.08 ± 0.67) (Fig. 2A). Next, to study the effect of co-culture time on microglia polarization, we co-cultured BMECs exosome and microglia for 6, 12, 24, 48, and 72 h, respectively, and then detected IL-1 β (secreted by M1 microglia) and IL-10 (secreted by M2 microglia) by ELISA (Additional file 1: Fig. S3). Compared with co-culture at 6 h, IL-1β level was enhanced after co-culture at 12, 24, 48, and 72 h in OGD-BMECs-exosomes and OGD-BMECs-NC inhibitor-exosomes groups while decreased in normal-BMECs-exosomes and OGD-BMECs-miR-3613-3p inhibitor-exosome groups. Compared with co-culture at 6 h, IL-10 level was decreased after co-culture at 12, 24, 48, and 72 h in OGD-BMECs-exosomes and OGD-BMECs-NC inhibitor-exosomes groups while increased in normal-BMECs-exosomes and OGD-BMECs-miR-3613-3p inhibitor-exosome groups, whereas IL-1β and IL-10 levels had no significant change between co-culture at 24, 48, and 72 h. The results showed that IL-1β and IL-10 levels are related to co-culture time with exosomes. However, the changes in the level of IL-1β and IL-10 were not significant after co-culture for 24 h. Therefore, the experiment was carried out at 24 h co-culture. The PKH 67 fluorescently labeled exosomes were co-cultured with HMC3 cells. Fluorescence microscopy revealed that exosomes were phagocytosed by HMC3 (Fig. 2B). In addition, miR-3613-3p expression in HMC3 was measured after BMEC-exosome co-culture (Fig. 2C). miR-3613-3p expression showed no prominent change between the control and normal BMEC-exosome co-cultured groups (1 ± 0.02 versus 0.96 ± 0.05). miR-3613-3p expression was prominently higher in the OGD-BMEC-exosome co-culture group than in the normal-BMEC-exosome co-culture group (11.8 ± 0.12 versus 0.96 ± 0.05), which was prominently reduced in the OGD-BMECs-miR-3613-3p inhibitor co-cultured group than that the OGD-BMECs-NC inhibitor co-cultured group (3.25 ± 0.11 versus 11.58 ± 0.29). Taken together, these results indicated that BMEC exosomal miR-3613-3p was transferred into HMC3.

### BMEC exosomal miR-3613-3p promotes microglia M1 polarization via the inhibition of the RC3H1 protein

**Fig. 3:**
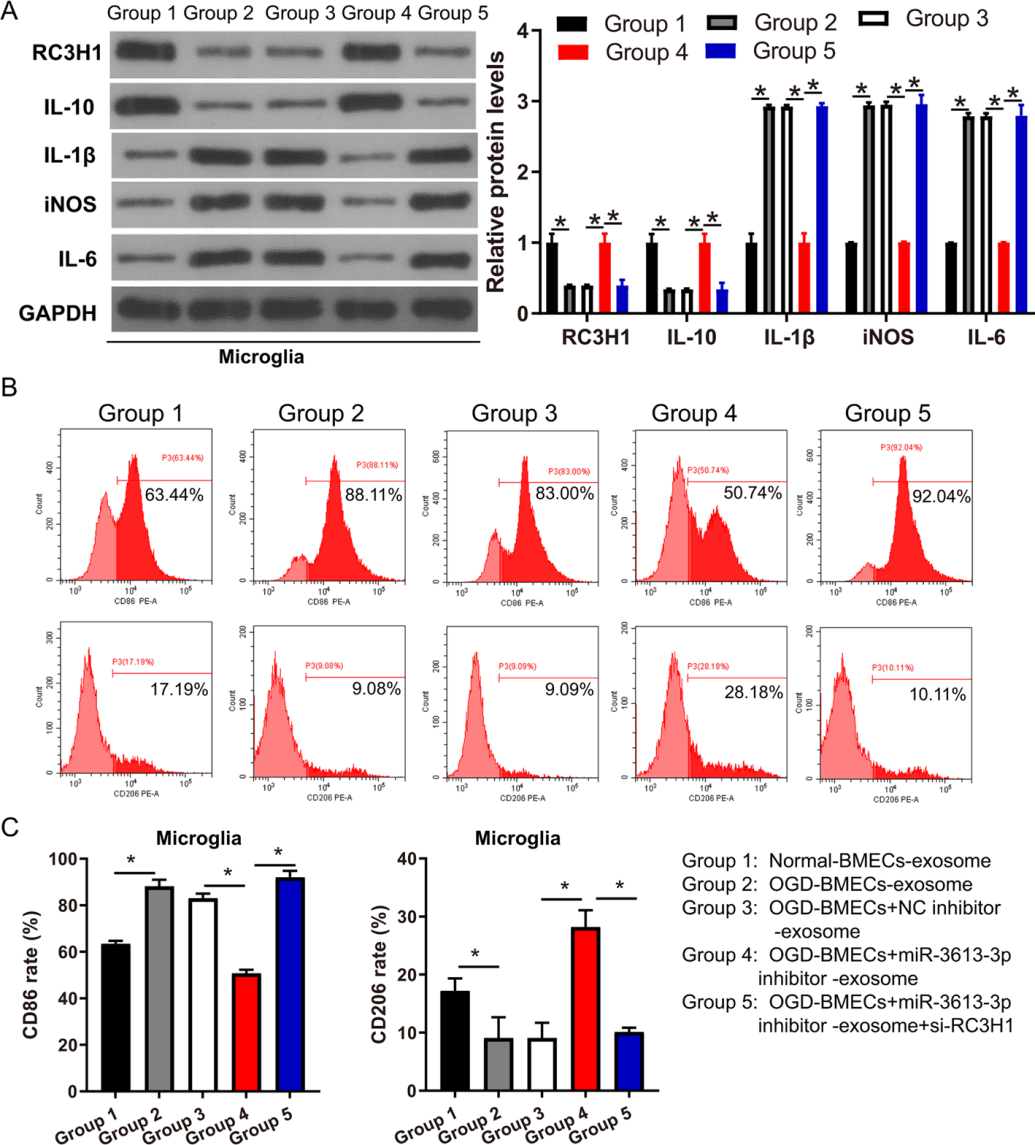
BMEC exosomal miR-3613-3p promotes microglia M1 polarization via inhibiting RC3H1 expression. **A** Protein levels were measured by western blot. The relative protein level is shown. **B** After co-culture with BMEC exosomes, M1 (CD86) and M2 microglia (CD206) were detected by flow cytometry. Apoptotic representative images are shown. **C** The rate of M1 (CD86) and M2 microglia (CD206) is shown. Differences were analyzed using ANOVA, followed by post-hoc Dunnett’s multiple comparisons test. *p < 0.05

To explore the regulatory mechanism of miR-3613-3p in microglia, target genes were analyzed using TargetScan, miRBD, and RNAinter. The results revealed 23 common genes via three website analyses (Additional file 1: Fig. S4A). Among these 23 genes, *RC3H1* is prominently associated with macrophage polarization [34]. In addition, potential binding sites for miR-3613-3p and *RC3H1* 3′ UTR were predicted using TargetScan (Additional file 1: Fig. S4B). The double luciferase assay showed that the relative intensity of fluorescence in the *RC3H1* 3′ UTR + miR-3613-3p group was significantly lower than that in the *RC3H1* 3′ UTR + NC group, suggesting that miR-3613-3p can bind to the *RC3H1* 3′ UTR. However, the relative intensity of fluorescence between mut-*RC3H1* 3′ UTR + miR-3613-3p and mut-*RC3H1* 3′ UTR + NC groups showed no prominent change, suggesting that miR-3613-3p can not bind to the mutational *RC3H1 *3′ UTR (Additional file 1: Fig. S4C). To explore the function and mechanism of BMEC exosomes in microglial polarization, microglia were treated with BMEC exosomes. Compared with the normal-BMEC-exosome group, the RC3H1 protein levels were prominently decreased in the OGD-BMEC-exosome group (Fig. 3A). Compared with the OGD-BMECs + NC inhibitor-exosome group, the RC3H1 protein levels were prominently increased in the OGD-BMECs + miR-3613-3p inhibitor-exosome group (Fig. 3A). However, transfection of si-RC3H1 to HMC3 cells (OGD-BMECs + miR-3613-3p inhibitor-exosome + si-RC3H1 group) reversed the effect of OGD-BMECs + miR-3613-3p inhibitor exosomes on RC3H1 protein levels (Fig. 3A). In addition, compared with the normal-BMEC-exosome group, the markers of M1 microglia, including IL-1β, iNOS, and IL-6, were prominently increased, whereas the marker of M2 microglia, IL-10, was prominently decreased in the OGD-BMEC-exosome group (Fig. 3A). Compared with the OGD-BMECs + NC inhibitor-exosome group, IL-1β, iNOS, and IL-6 were prominently decreased, whereas IL-10 was prominently increased in the OGD-BMECs + miR-3613-3p inhibitor-exosome group (Fig. 3A). However, the inhibition of RC3H1 expression (OGD-BMECs + miR-3613-3p inhibitor-exosome + si-*RC3H1* group) in HMC3 reversed the effect of OGD-BMECs + miR-3613-3p inhibitor-exosome on IL-1β, iNOS, IL-6, and IL-10 expression (Fig. 3A). Furthermore, M1 (CD86) and M2 microglia (CD206) showed the same trend as the western blotting results (Figs. 3B, C). OGD-BMECs exosome increased CD86 rate (88.11 ± 2.84%) while reducing CD206 (9.08 ± 3.58%) rate compared with normal-BMEC-exosome (CD86 rate: 63.44 ± 2.84% and CD206 rate: 11.19 ± 2.17%). In addition, OGD-BMECs + miR-3613-3p inhibitor-exosome treatment reduced CD86 rate (50.74 ± 1.59%) while increasing CD206 (28.18 ± 2.59%) rate compared with OGD-BMECs + NC inhibitor-exosome group (CD86 rate: 83.00 ± 2.10% and CD206 rate: 9.09 ± 2.14%), and CD86 rate (92.04 ± 2.74%) and CD206 rate (10.11 ± 0.75%) in OGD-BMECs + miR-3613-3p inhibitor-exosome + si-*RC3H1* group were reversed.

### BMEC exosomal miR-3613-3p reduces neuronal survival via the regulation of microglia M1 polarization

**Fig. 4:**
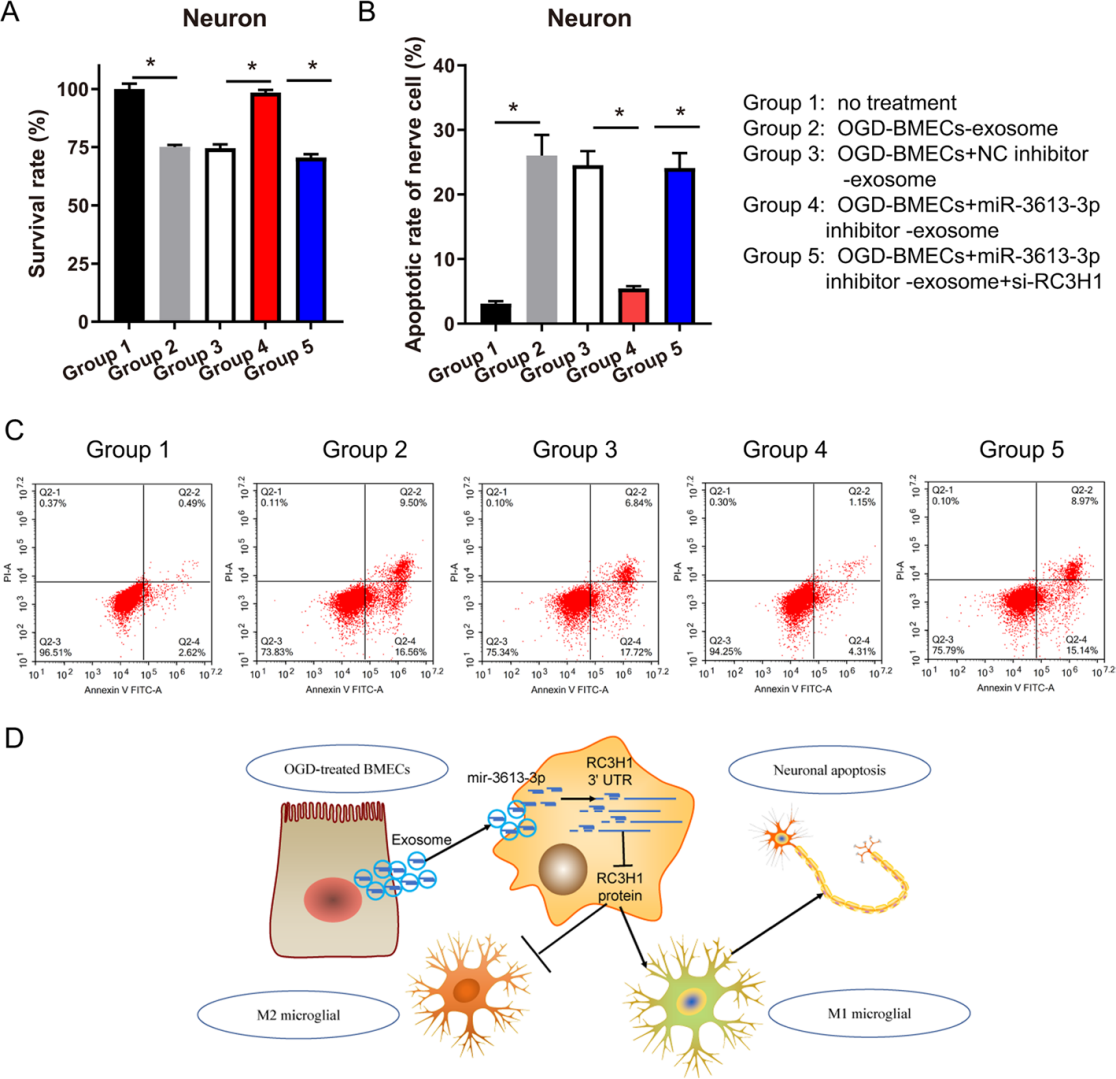
Microglia M1 polarization regulated by BMEC exosomes reduces neuronal survival. **A** The proliferation of neurons was analyzed by MTS. The survival rate as a percentage of the OD value of the other groups and the OD value of the no-treatment group. **B** The apoptotic rate of neurons was shown. **C** After co-culture with microglia, neuron apoptosis was detected by flow cytometry using Annexin V-FITC/PI apoptosis detection kit. Apoptosis representative images are shown. **D** Diagram of mechanism. Differences were analyzed using ANOVA, followed by post-hoc Dunnett’s multiple comparisons test. *p < 0.05

To explore the function of exosome-treated microglia in neuronal survival, nerve cells were treated with BMEC exosome-treated microglia. The survival rate of the no-treatment group, the OGD-BMEC-exosome-treated microglia group, the OGD-BMECs + NC inhibitor-exosome-treated microglia group, the OGD-BMECs + miR-3613-3p inhibitor-exosome-treated microglia group, and the OGD-BMECs + miR-3613-3p inhibitor-exosome-treated si-*RC3H1*-microglia group was 100.00 ± 2.324, 75.16 ± 0.788, 74.61 ± 1.652, 98.43 ± 1.178, and 70.54 ± 1.442, and the apoptosis rate was 3.11 ± 0.37, 26.05 ± 3.15, 24.56 ± 2.16, 5.46 ± 0.35, and 24.11 ± 2.31, respectively. Compared with the no-treatment group, the survival of nerve cells was prominently reduced, while the apoptosis of nerve cells was prominently increased in the OGD-BMEC-exosome-treated microglia group (Fig. 4A, B). Compared with the OGD-BMECs + NC inhibitor-exosome-treated microglia group, the survival of nerve cells was prominently increased, while the apoptosis of nerve cells was prominently reduced in the OGD-BMECs + miR-3613-3p inhibitor-exosome-treated microglia group (Fig. 4A, B). Compared with the OGD-BMECs + miR-3613-3p inhibitor-exosome-treated microglia group, the survival of nerve cells was prominently decreased, while the apoptosis of nerve cells was prominently increased in the OGD-BMECs + miR-3613-3p inhibitor-exosome-treated si-*RC3H1*-microglia group (Fig. 4A, B). The apoptosis of nerve cells detected using Annexin V-FITC/PI apoptosis detection kit is shown in Fig. 4C.

## Discussion

Exosomes, enriched and transferred mRNA, proteins, miRNAs, and other noncoding RNA are important vehicles between cells and have received extensive attention for stroke treatment [35, 36]. Hence, exosomes from normally cultured and OGD-cultured BMECs were collected, and differentially expressed miRNAs were analyzed. In the exosomes of OGD-treated BMECs, 205 miRNAs were prominently reduced, while 49 miRNAs were prominently enriched. RT-qPCR verification further revealed that miR-3613-3p was most prominently enriched in exosomes from OGD-treated BMECs. The brain microvascular network is severely damaged after ischemic stroke [10, 37]. Previous studies have suggested that BMEC injury can affect neuronal survival by modulating immune responses through the microenvironment [14, 21]. Here, we found that miR-3613-3p knockdown enhanced cell survival, migration, and angiogenesis in OGD-treated BMECs, suggesting that miR-3613-3p can ameliorate the loss of BMEC function caused by OGD.

Recently, the studies showed that the conditioned medium of damaged BMECs promotes microglial inflammation secretion [38, 39]. However, the regulating the signal axis has not been established. In addition, miRNAs that enter the exosomes through an active sorting mechanism can be absorbed by receptor cells, then regulated the biological function of receptor cells [40]. miRNAs such as miR-146a-5p, miR-192-5p, miR-146a-5p, and miR-34a in exosomes can affect the M1/M2 polarization of microglia [41, 42]. Here, we found that exosomal miR-3613-3p in OGD-treated BMECs can be transferred to microglia and promote the polarization of glioma cells towards the M1-type. Microglia are important immune-regulatory cells in the central nervous system that regulate stroke through different polarizations; M1-type polarization induces neuroinflammation and neurotoxicity, whereas M2-type polarization plays the opposite role, thereby exerting a neuroprotective effect and improving the prognosis of stroke [43]. As expected, M1-type microglia induced by exosomal miR-3613-3p in OGD-treated BMECs promoted neuronal apoptosis and inhibited cell survival. These results suggest that exosomal miR-3613-3p in BMECs increases neuronal apoptosis by inducing microglial M1 polarization.

Exosomes can regulate the translation of miRNA-targeted genes in microglia by transmitting miRNA. Exosomal miR-146a-5p inhibited IRAK1/TRAF6 expression to reduce microglial polarization after ischemic stroke [42]. In this study, we confirmed that miR-3613-3p binds to the *RC3H1* 3′ UTR to inhibit RC3H1 protein levels in microglia. RC3H1, also known as ROQUIN, is an E3 ubiquitin protein. Defective RC3H1 affects macrophage, T-cell, and B-cell functions and induces innate and adaptive immune diseases [44]. RC3H1 knockdown promotes macrophage polarization towards M1 but not towards M2 in hepatic ischemia–reperfusion injury [34]. RC3H1 overexpression inhibits TNF-α expression (M1 polarization) in decidual macrophages [45]. Similar to the above findings, we found that exosomal miR-3613-3p can inhibit RC3H1 protein level to promote microglial M1 polarization.

However, there are four limitations to the present study. Firstly, we did not carry out the target protector experiment to prove that RC3H1 is the only target of miR-3613-3p regulation. Therefore, miR-3613-3p may also play the important role by regulating other targets. Next, 5 × 10^9^ granules or 50–100 µg exosomes can regulate the polarization of microglia in vivo [42, 46, 47]. However, the exosome concentration of OGD-treated BMECs that can regulate the microglia polarization needs further exploration in vivo. Furthermore, The direct regulatory role of BMECs and nerve cells is unclear. Whether BMECs can regulate the death or regeneration of nerve cells through exosomes remains to be further studied. Finally, whether other components in exosomes play an important role is also worth further study.

## Conclusions

miR-3613-3p knockdown enhanced the survival, migration, and angiogenesis of OGD-treated BMECs. Interfering with miR-3613-3p expression in BMECs reduced the enrichment of miR-3613-3p in exosomes and its transport into microglia and promoted the polarization of microglia toward M2 and the reduction of neuronal apoptosis by partly increasing RC3H1 protein. Exosomal miR-3613-3p plays an important role in the NVU and may be a potential target for stroke therapy (Fig. 4D).

## Supplementary Information

**Additional file 1: Appendix S1. **Survey questions used in the analysis. **Appendix S2. **Beta coefficients for business expansion. **Appendix S3. **Beta coefficients for business selling. **Appendix S4. **Variance inflation factor (VIF) values.

## Data Availability

The datasets used and/or analyzed during the current study are available from the corresponding author on reasonable request. Different miRNAs between exosome from the culture medium of OGD-treated and normally treated BMECs were analyzed via the miRNA GeneChip assay and shown in GSE224961 (https://www.ncbi.nlm.nih.gov/geo/query/acc.cgi?acc=GSE224961).

## Abbreviations

BMEC: Brain microvascular endothelial cell
OGD: Oxygen–glucose deprivation
miRNA: MicroRNA
RT-qPCR: Real-time polymerase chain reaction
NVU: Neurovascular unit
RC3H1: Ring finger and CCCH-type domains 1
