# Supplementary material for: Exosomal Mir-3613-3p derived from oxygen–glucose deprivation-treated brain microvascular endothelial cell promotes microglial M1 polarization
Source: Cell Mol Biol Lett. 2023 Mar 5;28:18. doi: 10.1186/s11658-023-00432-1 (PMC9985860; doi:10.1186/s11658-023-00432-1)
Supplement: Supplementary file 1 — Additional file 1: Appendix S1. Survey questions used in the analysis. Appendix S2. Beta coefficients for business expansion. Appendix S3. Beta coefficients for business selling. Appendix S4. Variance inflation factor (VIF) values. [file 11658_2023_432_MOESM1_ESM.pdf]

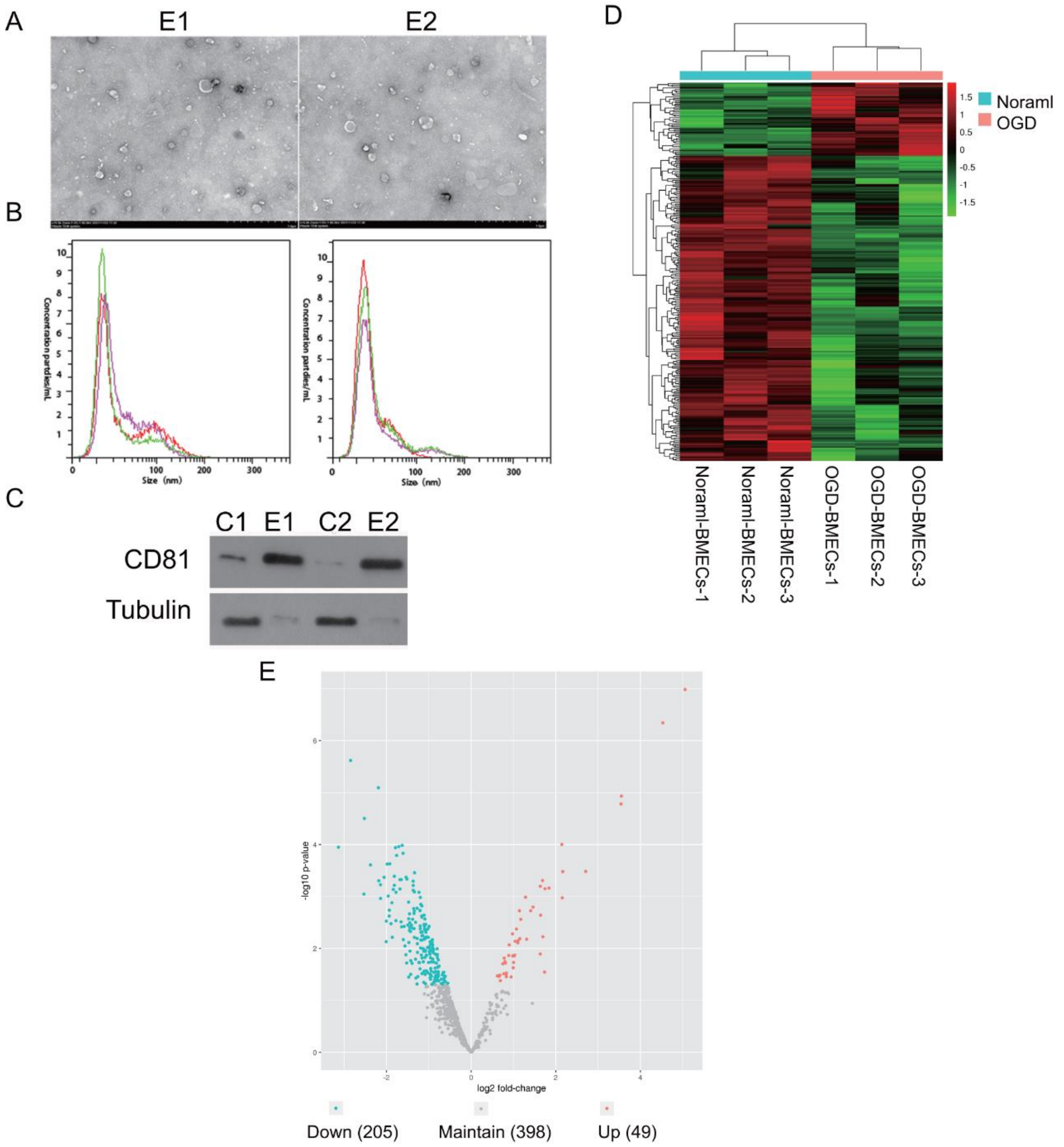

Supplementary Figure 1 Abnormal miRNAs were found by miRNA high-throughput sequencing in the exosome of OGD-treated BMECs.

(A) Exosome diameters were examined by NTA detection. (B) Morphology of the exosomes was observed by an electron microscope. (C) The exosome marker CD81 and cell tubulin protein were detected by western blot. (D) Heat map showing abnormal miRNAs in the exosome of OGD-treated BMECs (OGD group) and normally treated BMECs (normal group). (E) Abnormal miRNAs were shown in the Volcano plot. Green and red plots indicate low and high expression, respectively. E1 and E2: the exosome of OGD-treated BMECs and normally treated BMECs; C1 and C2: the OGD-treated BMECs and normally treated BMECs.

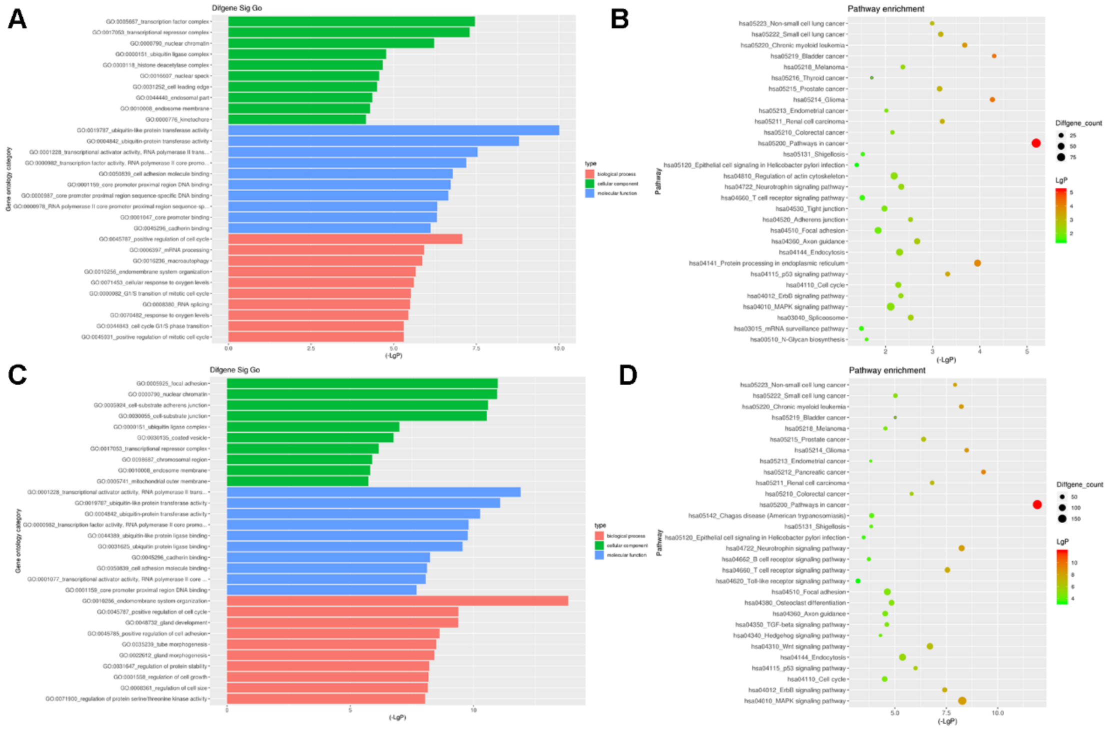

Supplementary Figure 2 Bioinformatic analysis was conducted using the enrichment analysis of GO and KEGG terms for abnormal miRNAs.

(A) GO analysis of abnormally down-regulated miRNAs. (B) KEGG analysis of abnormally down-regulated miRNAs. (C) GO analysis of abnormally up-regulated miRNAs. (D) KEGG analysis of abnormally up-regulated miRNAs.

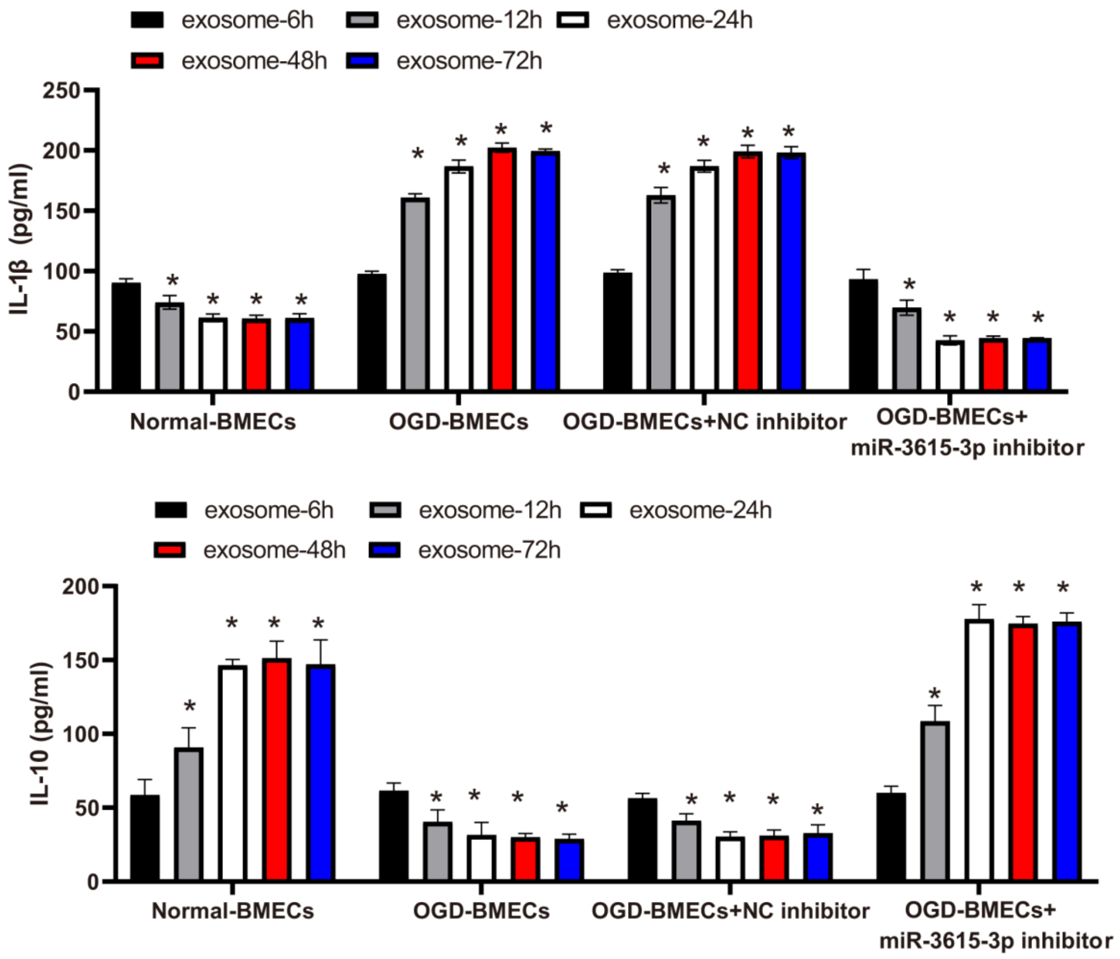

Supplementary Figure 3 BMECs exosomal miR-3613-3p promotes IL-1β level while inhibits IL-10 level in microglia. IL-1β and IL-10 levels were measured by ELISA. *P<0.05, vs exosome-6h.

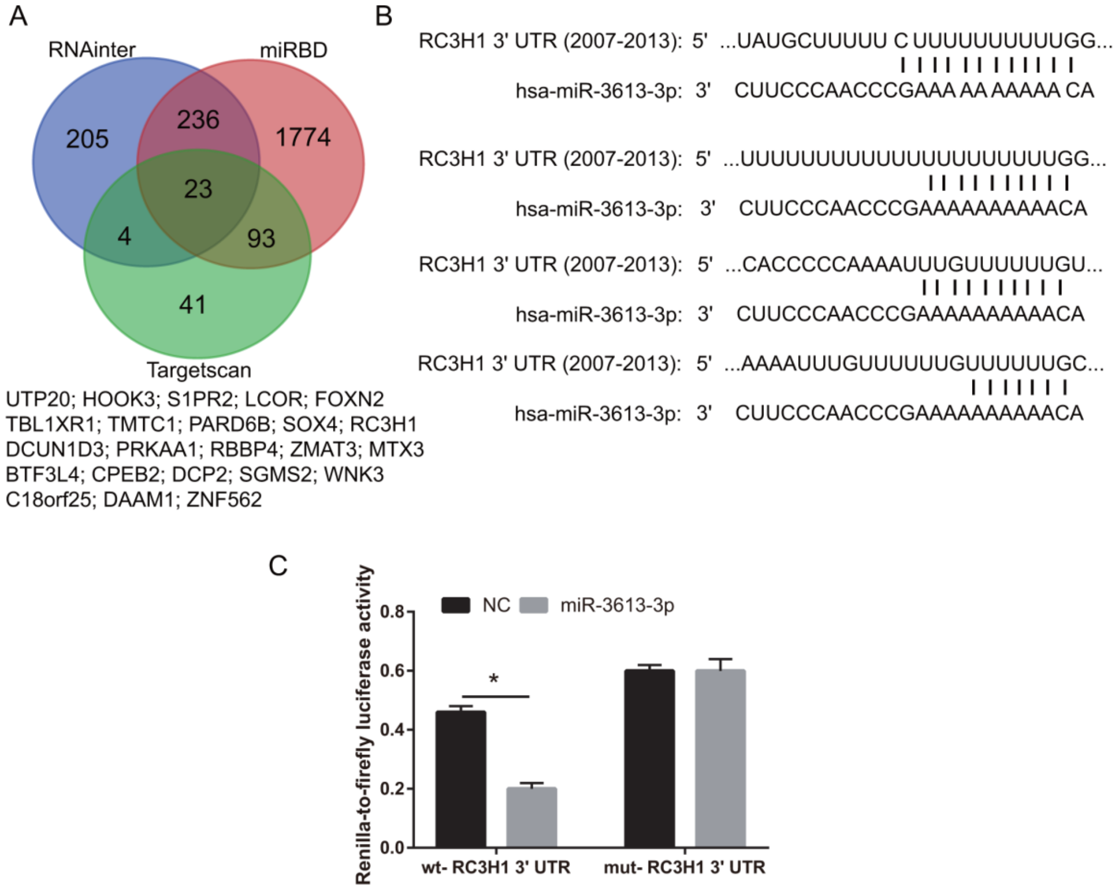

Supplementary Figure 4 RC3H1 was the potential target of miR-3613-3p in microglia.

(A) The potential target of miR-3613-3p was analyzed by targetscan, miRBD, and RNAinter websites. (B) Four bound sites between miR-3613-3p and RC3H1 3' UTR were found on the targetscan website. (C) The double luciferase experiment confirmed the existence of binding of miR-3613-3p and RC3H1 3' UTR. *P<0.05.
